# The Quantification of IgG Specific to α-Gal Could Be Used as a Risk Marker for Suffering Mammalian Meat Allergy

**DOI:** 10.3390/foods11030466

**Published:** 2022-02-04

**Authors:** Alejandro Joral, Nahikari Azketa, Patricia Sanchez, Ainara Vélez-del-Burgo, María-Ascensión Aranzabal-Soto, Susana Lizarza, Jorge Martínez, Idoia Postigo

**Affiliations:** 1 Servicio de Alergología, Hospital Universitario Donostia, 20014 San Sebastián, Spain; ALEJANDRO.JORALBADAS@osakidetza.eus (A.J.); SUSANA.LIZARZAMENDIZABAL@osakidetza.eus (S.L.); 2 Parasitology and Allergy Research Group, Lascaray Research Institute, Department of Immunology, Microbiology, and Parasitology, Faculty of Pharmacy, The University of Basque Country, 01006 Vitoria, Spain; nahikari.azketa@ehu.eus (N.A.); patricia.sanchez@ehu.eus (P.S.); ainara.velezdelburgo@ehu.eus (A.V.-d.-B.); jorge.martinez@ehu.eus (J.M.); 3 OSI Goierri Alto Urola, 20700 Zumárraga, Spain; MARIASCENSION.ARANZABALSOTO@osakidetza.eus

**Keywords:** α-Gal, food allergy, red meat allergy, tick, sIgG, sIgE

## Abstract

The alpha-Gal Syndrome is a delayed meat allergy characterized by the presence of sIgE against α-Gal epitope. It is known that the α-Gal present in tick saliva induces the sensitization to this epitope ending in the production of sIgG and sIgE to α-Gal. It could be considered that the more times a person is bitten by tick species, the higher the probability of making the switch from sIgG to sIgE to α-Gal and developing allergy, but it is no clear when the switch occurs. To determine the likelihood that a subject bitten by ticks but without AGS be at risk of developing this allergy, we quantified the levels of sIgG to α-Gal by an automated system (ImmunoCap). To stablish a cut-off value for sIgG to α-Gal, a receiving operating curve (ROC) was constructed. The statistical analysis demonstrated that the risk of suffering AGS in individuals bitten by ticks was 35% when the sIgG to α-Gal was greater than or equal to 40 µg/mL. Our data indicate that the sIgG values against α-Gal could be used as a prognostic marker for developing mammalian meat allergy.

## 1. Introduction

Mammalian meat allergy, also referred to as alpha-Gal syndrome (AGS), is a special kind of delayed food allergy to the carbohydrate moiety galactosyl-α-1,3-galactose (α-Gal) [1,2]. Clinically, α-Gal allergy is characterized by reactions to mammalian meat and innards, including beef, pork, and lamb, which occur 3–6 h following meat consumption [3,4,5,6]. The symptoms of this allergy are variable ranging from abdominal pain and diarrhea to urticaria episodes and anaphylaxis [7]. The α-Gal carbohydrate is present in tissues from several mammalians except humans [8,9] and it has been demonstrated in the salivary glands of several tick species [10,11,12]. It is assumed that the main cause of sensitization in AGS is the recurrent tick bite [13,14] and, the most prevalent tick species in each continent could be responsible for this allergy [15,16].

In Europe, the frequency of positivity of sIgE to α-Gal has been reported to be 5.5% in Denmark [17], 15.7% in a representative Spanish cohort [18] and 24.7% in a rural area in northeast Italy [19]. In the United States (U.S.), more than 5000 cases have been described [20]. In addition, new cases that are not directly related to meat intake are being described, e.g., immediate allergic reactions in α-Gal positive patients to common vaccines, which contain mammalian-derived gelatin [21]. 

The knowledge about this syndrome began in the southeastern of the U.S. with the use of cetuximab, a mouse–human chimeric antibody (Ab) for the treatment of cancer [22]. Although the clinical trials demonstrated a low risk of allergy to the drug, these patients from a specific U.S. geographic area presented severe drug allergic reactions, higher than the expected [23]. Further investigations demonstrated that the patients who experienced hypersensitivity reactions had pre-existing IgE antibodies (Abs) that bound to the α-Gal carbohydrate moiety found in the murine portion of the chimeric Ab [23]. Therefore, they should have been sensitized in some way. At the same time and, in the same southern region of the U.S., physicians perceived an increase in cases of healthy individuals experiencing urticaria, angioedema or anaphylaxis several hours after consuming red meat [24]. In many cases, the individuals who experienced these hypersensitivity responses had a history of consuming meat for decades with no adverse reaction [25]. Further work revealed that α-Gal-specific IgE contributed significantly to the allergic response to red meat in these individuals [26]. Later on, the research groups realized that both cetuximab-induced hypersensitivity and meat allergy cases were restricted to the same geographical area where the lone star tick was prevalent [26]. In Australia, the investigations revealed that a large number of patients with meat allergy also had a history of tick bites [27] and, in 2007, the first report of the capacity of ticks to induce mammalian red meat allergy was published [28]. Since then, this syndrome is becoming a global problem and an increasing number of cases are being reported from almost all continents [29,30,31,32,33].

Old World monkeys, apes, and humans do not express the α-Gal containing oligosaccharide because, in humans, the α-1,3-galactosyltransferase (α-1,3GT) is expressed in an enzymatically inactive form [34]. That is the reason why all immunocompetent humans can express in a “natural” way anti-α-Gal Abs [34] against the α-Gal epitopes. It is though that the antigenic source for developing the anti-α-Gal Abs is the continuous exposition to this moiety present in the outer membrane of bacteria from the intestinal microbiome [35]. Thus, when foreign α-Gal antigens enter the body, the anti-α-Gal B cells are stimulated and can produce large amounts of high-affinity anti-α-Gal Abs [36]. It is estimated that one percent of human-circulating B-lymphocytes are capable of producing the natural anti-α-Gal Abs, mainly IgM and IgG isotypes [37].

The AGS-patients are characterized by elevated values of specific IgE and IgG Abs against α-Gal epitope [38,39,40,41] and, probably, most AGS-patients who had tolerated red meat for many years could have sensitized to α-Gal through tick bites [40]. The α-Gal epitope has been identified in the salivary glands [42] and cement [43] of several tick species, including the most prevalent hard tick in Europe: *Ixodes ricinus* [10,12]. The mechanism by which tick bites sensitize individuals to this epitope and, as a result, induce the meat allergy is not clear yet. Given that α-Gal exposure alone does not induce an IgE response [44], there must be a sensitization process, maybe by means α-Gal antigen present on tick salivary proteins or through the presence of immunomodulatory factors in tick saliva, such as prostaglandin E2 (PGE2) [45]. Maybe, the continuous exposition to tick bites could induced in exposed individuals the switch of pre-existing anti-α-Gal-IgG Abs to sIgE to α-Gal helped by immunomodulatory agents, such as PGE2.

There are several works on the Ab titers of the different Ig isotypes and IgG subclasses expressed in both AGS patients and healthy donors, including their relationship to the blood group of the individuals [46,47] and, the published data on the concentration of IgG to α-Gal in normal human serum are controversial depending on the methodology used for quantification. [37,48,49,50]

The objective of our study was to quantify, by means of an automated and standardized method, the levels of sIgG to α-Gal in four different groups of adult subjects: clinical diagnosed AGS-patients, atopic subjects, healthy donors, and in a group of subjects bitten by ticks but who had not developed AGS. The aim was to stablish a sIgG to α-Gal cut-off value. Before, we analyze the likelihood that a subject bitten by ticks, with sIgG to α-Gal but without AGS, could be at risk of developing this allergy.

## 2. Materials and Methods

### 2.1. Study Design and Ethical Approval

The study protocol was approved by the Ethics Committee of the Hospital BioDonosti- Osakidetza, in accordance with The Code of Ethics of the World Medical Association, Declaration of Helsinki. 

Two hundred people living in the Basque Country community (North of Spain) were contacted through social networks (Facebook) to participate in the study and, fifty subjects voluntarily accepted to participate. Forty-six people from the fifty volunteers were recruited through a survey for this cross-sectional study. The age range was 20–60 years. All selected individuals declared to be bitten by ticks, but none reported clinical AGS-related symptoms or had been clinically diagnosed with AGS. This group was labeled as the risk population group. The community health centers give each individual a medical flyer for blood collection and written informed consent was obtained from all of the individuals. The frozen serum samples were sent to the Parasitology and Allergy Laboratory-Lascaray Research Center-UPV/EHU for analysis. The basic epidemiological information, such as age, gender, habitat, presence of allergies and some items about the meat consumption behavior or the contact with animals (dog, cat) of each subject in the risk-population group, were recorded by survey.

Sera from 15 adult (20–60 years) patients clinically diagnosed with AGS were included in the study as positive control group. The AGS diagnostic was based on case history, positive skin prick tests, and the presence of α-Gal-specific IgE in the serum of these patients in concentrations higher than 0.35 kU_A_/L. All subjects mentioned being bitten by ticks. This group was labeled as AGS patients.

Sera from 108 healthy individuals who did not report any allergic symptoms and showed no allergen-specific IgE Ab (ImmunoCap ISAC. Thermo Fisher Scientific, Waltham, MA, USA) were selected from our serum collection (National Register of Biobank Serum Collections, code C.0002774; Instituto de Salud Carlos III, Ministry of Economy and Competitiveness/Lascaray Research Center, University of the Basque Country, Vitoria, Spain). All the samples belonged to adult people living in urban areas. None of them mentioned tick bites. This group was considered as the healthy population control group.

Finally, 64 sample sera from atopic adult subjects constituted the allergic control group. None of them mentioned being bitten by ticks and all of them came from urban areas.

### 2.2. Determination of sIgE Antibodies against Different Allergens

To assess the profile of sensitization in the risk and atopic populations, the sIgE against a panel of 112 allergens, including grass pollen, tree pollen, mites, fungi, food allergens, animal dander, insect venom and CCDs, was measured by Immuno Solid-phase Allergen Chip (ImmunoCAP ISAC 112. Phadia AB, Uppsala, Sweden) following the manufacturer’s instructions [51]. This technique is a multiplex assay based on component-resolved diagnosis [52]. The allergen components are spotted in triplets and covalently immobilized on a polymer-coated slide. Briefly, 30 μL of serum samples was added to each microarray and incubated at room temperature for 120 min. After washing, 30 μL fluorescence-labeled antihuman IgE Abs were added. Following incubation for 30 min, unbound labeled Abs were removed by washing, and fluorescence was measured with a laser scanner. The results were evaluated using Phadia Microarray Image Analysis (MIA) software. ImmunoCAP ISAC is a semi-quantitative test and results are reported in ISAC Standardized Units (ISU) giving indications of specific IgE Ab levels within a measuring range of 0.3–100 ISU-E. The ISU-E are standardized to ImmunoCAP Specific IgE units [51].

In this study, all samples in the range of 0.3–100 ISU-E were considered positives and the results were expressed as the percentage of positives to each allergen in the studied populations.

### 2.3. Quantification of sIgE and sIgG Specific Antibodies against the α-Gal Epitope 

In all samples, the sIgE and sIgG Ab levels against α-Gal epitope were quantified by fluoro-enzyme-immunoassay (FEIA) using an automated system (ImmunoCap, Thermo Fisher Scientific), according to the manufacturer’s instructions. The cut-off value stablished by the manufacturer for the sIgE Ab was 0.35 kU_A_/L.

A receiver operating characteristic (ROC) curve was constructed to establish the sIgG cut-off value to α-Gal in order to differentiate the patients bitten by ticks who developed AGS from the control groups (atopic subjects and healthy individuals) not bitten by ticks [53].

### 2.4. Statistical Analysis

Data were entered in Graph Pad Prism 7.0 for statistical analysis. Statistical differences among populations were determined using the no parametric Kruskal–Wallis test and Dunn’s multiple comparisons. Differences were considered statistically significant for *p* < 0.05 (95% CI).

A ROC curve was plotted using the GraphPad Prism v 7.0 software and the area under the curve was calculated to quantify the accuracy of the test.

To assess the association between the likelihood of suffering AGS and the sIgG levels to α-Gal, the contingency analysis was performed using the Fisher exact test. A *p* ≤ 0.05 was considered statistically significant. The relative and attributable risk were calculated by Koopman asymptomatic score and odds ratio by the Baptista–Pike method with 95% CI.

## 3. Results

### 3.1. Clinical and Demographic Data of Participants

#### 3.1.1. AGS Patients

The 15 AGS patients had experienced meat-induced symptoms occurring 3–7 h after ingestion that comprised anaphylaxis (53.3%), acute urticaria (13.3%), and recurrent urticaria (33.3%). The IgE specific to α-Gal ranged from 12.3 to >100 kU_A_/L, the average being 62.5 kU_A_/L (Table 1).

#### 3.1.2. Risk-Population Group

The 78% of the risk population subjects declared to be daily meat consumers (mainly beef and/or pork) and the 9% declared suffering from some intolerance directly related with meat consumption. The basic epidemiological information of participants in risk-population group (*n* = 46) are given in Table 2. A total of 83% of the participants live in an urban environment and 85% declared animal contact (dogs and cats). A total of 30% declared suffering from chronic diseases, such as Lyme, asthma, arthritis, and fibromyalgia. A total of 9% declared other diseases, such as anxiety, psoriasis, hypothyroidism, hyperactivity, hearing loss, or acute pyelonephritis.

#### 3.1.3. Profiling of sIgE Antibodies in Risk- and Atopic Population Groups

Data on the atopic condition of the risk and atopic populations are summarized in Table 3. A total of 54% of the risk population declared having some type of allergy. The data on IgE specific to the array of studied allergens (ImmunoCap ISAC. Thermo Fisher Scientific) demonstrated that Der 1 and Der 2 mite allergens (30%), grass pollen (Phl p1) (15%), cat uteroglobin (Fel d 1) (13%), and lipocalins from cat, dog and mouse (12.9%) were the allergens implicated in the risk-population atopic condition. Sensitization to food and molds allergens was not demonstrated. One individual showed IgE specific to cross-reactive carbohydrates determinants (Mux F3-bromelain) and two people to wasp venom (Pol d 5- antigen 5).

In the atopic group, the sensitization to grass pollen (53%) was the most prevalent followed by the sensitization to tree pollens (olive and cypress) and to Der 1 and Der 2 mites allergens (30 and 37%, respectively). Sensitization to food allergens, such as apricot (20%), hazel (7.8%), kiwifruit (7.8%), shrimp (4.6%) and egg (4.6%), was demonstrated and to fungal allergens, such as *Alternaria alternata* (12.5%) and *Aspergillus fumigatus* (7.8%). Finally, 32% of the atopic subjects showed sensitization to cat epithelium, with sensitization to cat uteroglobulin (Fel d 1) being the most prevalent (26%).

### 3.2. Quantification of sIgE and sIgG Antibodies against the α-Gal Epitope

**Figure 1:**
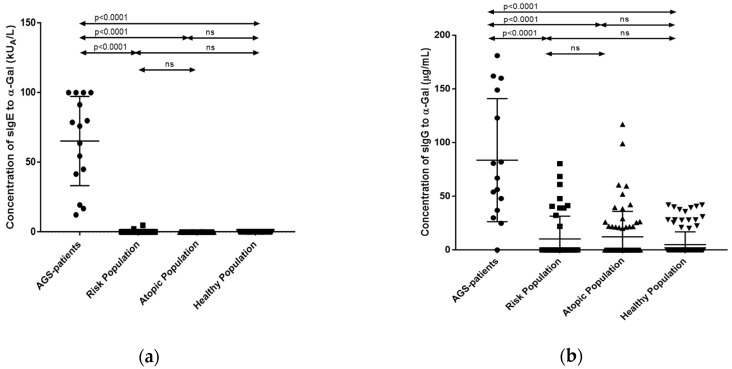
Concentrations of the sIgE (**a**) and sIgG (**b**) antibodies specific to α-Gal in the studied populations: AGS patients (circles) ; Risk Population (squares); Atopic Population (triangles up-ward); Healthy Population (downward).

The sIgE mean value against α-Gal in the AGS patients was 62.5 ± 8.3 kU_A_/L (Figure 1a). No positive values (≥0.35 kU_A_/L) were demonstrated in atopic and healthy populations (mean values 0.014 ± 0.003 kU_A_/L and 0.013 ± 0.001 kU_A_/L, respectively). Two participants (4.3%) in the risk-population group have the concentrations of sIgE to α-Gal 4.82 and 2.13 kU_A_/L, which was higher than the minimal values for the positive test >0.35 kU_A_/L). Statistically significate differences were only demonstrated among AGS patients and the risk-population group, the atopic population group, and the healthy population group (*p* < 0.001).

In 14 of 15 of AGS patients (93%), α-Gal-specific IgG Abs were found at concentrations between 25 and 190 µg/mL. The mean value obtained for this group was 83 ± 1.4 µg/mL) (Figure 1b).

A total of 21.7% of the subjects in the risk-population group (10/46), showed IgG specific to α-Gal (mean value: 10.3 ± 3.1 µg/mL). The 31.2% and the 15.7% of the atopic and healthy population, respectively, demonstrated sIgG against α-Gal epitope (mean values: 12.3 ± 2.9 µg/mL and 0.5 ± 1.1 µg/mL, respectively). Statistically significate differences were only demonstrated among AGS patients and risk-population group, atopic group, and the healthy population (*p* < 0.001).

**Figure 2:**
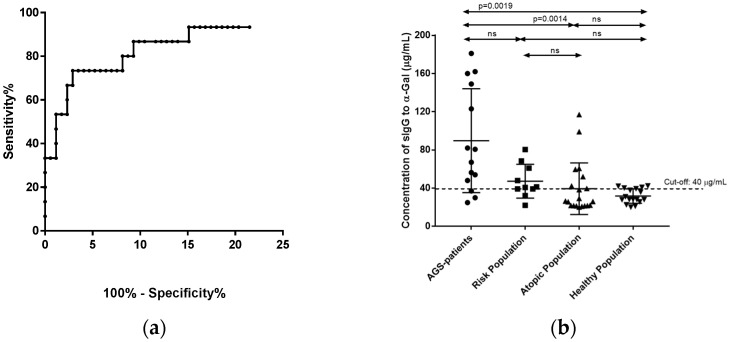
ROC curve (**a**) and distribution of the studied populations considering 40 µg/mL as sIgG to α-Gal cut-off value (**b**). AGS patients (circles) ; Risk Population (squares); Atopic Population (triangles upward); Healthy Population (downward).

A ROC was constructed to establish the sIgG Ab cut-off value against the α-Gal epitope (Figure 2a). The optimal cut-off point for specific IgG against α-Gal was obtained by ROC analysis (area under the curve: 0.931; standard error: 0.042; 95% confidence interval: 0.721 to 0.847; *p* < 0.001). The mean value plus SD (40 µg/mL) was chosen as the cut-off value (sensitivity, 86.7%; specificity, 92.6%). A new statistical analysis of the data was made according to the cut-off value (Figure 2b).

In this case, the statistical analysis showed no differences between the AGS patient group and the risk-population group included in this study (*p* > 0.999). Statistically significate differences were demonstrated among AGS patients and the atopic population and healthy population (*p* = 0.002 and *p* = 0.001, respectively). A contingency analysis was performed to assess the association between the likelihood of suffering AGS and the sIgG levels to α-Gal. The statistical analysis showed a likelihood of 35% of suffering AGS when the sIgG to α-Gal was greater than or equal to 40 µg/mL in people bitten by ticks. The risk for suffering AGS was increased by 17.5 times (CI 6.333–50.271) when sIgG values to α-Gal was greater than the cut-off value. Serum IgG specific to α-Gal was a significant predictor of AGS with an odds ratio of 27.2 (CI 8.153–81.190). The attributable risk was 33.5% (CI 0.175–0.526).

## 4. Discussion

Although the studies of the biochemical nature and function of α-Gal began more than two decades ago in xenotransplantation [54,55], it was in 2008 when Chung et al. published the anaphylaxis reaction to α-Gal epitope present in cetuximab, a chimeric monoclonal Ab used in the treatment of some cancer [18]. Commins et al., in 2008, demonstrated that patients with IgE specific to α-Gal epitope suffer from delayed anaphylaxis, angioedema, or urticaria after the consumption of red meat. In 2007, van Nunen et al. published the first report on the capacity of ticks to induce red meat allergy [28]. Since then, several authors have provided elegant proofs of the association between the injection of α-Gal present in tick saliva and cement with the development of mammalian meat allergy [56,57].

The association between tick bite reactions and red meat allergy in humans was described in Australia [57]. Before, different studies on red meat allergy have demonstrated that AGS culprit tick species are found in almost all continents [27]. Our study was carried out in people from the North of Spain (Basque Country), where the most prevalent ticks are *I. ricinus* and *Hemaphysalis punctate* [58]. All the clinically diagnosed AGS patients had been bitten by ticks, presented IgE specific to α-Gal and, almost all of them (14/15) presented IgG specific to α-Gal. In the same way, all the risk population subjects were bitten by ticks, but none of them was AGS diagnosed. However, two participants presented IgE specific to α-Gal positive values (4.82 kU_A_/L and 2.13 kU_A_/L) revealing the sensitization to the epitope without clinical symptoms. Mabelane et al. (2018) established that the α-Gal IgE value above which there was a 95% probability of meat allergy was 5.5 kU_A_/L [59]. These data explain, probably, that none one of these participants were referred for suffering red meat allergy, although other authors stablished the cut-off value at >0.54 kU_A_/L for sIgE [60]. No individuals in control groups (healthy and atopic population) showed IgE Abs specific to α-Gal. Nevertheless, all the studied groups demonstrated IgG Abs specific to this oligosaccharide moiety. Humans do not express the α-Gal carrying oligosaccharide because, in humans, α-1,3GT is expressed in an enzymatically inactive form [8,9]. The origin of this mutation occurred thousands of years ago and, it was probably an evolutionary step for humans in the defense against viruses, bacteria, and parasites carrying this oligosaccharide on its surfaces, such as *Trypanosoma* and *Leishmania* [61]. All immunocompetent humans can develop a strong immune response against the α-Gal epitope [33], which is considered as the only naturally abundantly expressed Abs in humans [8,9]. Hamanova et al. (2015) studied the kinetics for the formation of anti-α-Gal Abs (IgM, IgA, and IgG) in a group of infants along their first two years of life [62]. They demonstrated the transplacental transfer of the anti-α-Gal IgG Abs, which started to increase slowly with increasing age [63]. It is suggested that these Abs are produced at all human ages against the α-Gal epitopes present in the outer membrane of bacteria from the intestinal microbiome [34,35]. The continuous antigen stimulation by gut bacteria induces that as much as 1% of the human B cell population (memory B cells) in an individual is capable of producing anti-α-Gal Abs [36]. However, the data on the concentration of IgG to α-Gal in normal human serum are controversial depending on the methodology used for quantification. Galili et al. (1984) established that the level of anti-α-Gal Abs in healthy donors was 1% [8]; Yu et al. (1996) quantified by ELISA that the value was in the range of 0 to 15 µg/mL [27], and Tomlinson and Nussenzweig (1997) established this value between 0.25 and 0.5% of total Igs [48]; Obukhova et al. (2007) indicated that the level of Igs against α-Gal in healthy donors was about 10-fold less than the established by Galili et al. (1984) [49]. Recently, Zappe et al. (2021) published that the level of IgG anti-α-Gal in a commercial concentrate of human IgG is about 10% (IgG1 isotype) [50].

In our study, it was demonstrated that the AGS patients had higher statistically significant anti-α-Gal sIgG levels than the risk population and the control groups according to other authors [46,47]. These data were of interest, and we performed a ROC to establish an α-Gal IgG cut-off value to study the possibility of using anti-α-Gal IgG as an AGS prognostic marker. The statistical analysis of the re-arranged data demonstrated that the sIgG positive values at the risk population and AGS patients followed a similar distribution from the statistical point of view. Then, we calculated the probability that tick-bitten people with sIgG Ab levels to α-Gal greater than 40 µg/mL would develop AGS. The results indicated that the presence of sIgG Ab to α-Gal at levels greater than the cut-off value in serum was a risk factor for developing sIgE Ab to α-Gal greater than 0.35 kU_A_/L. This event was calculated to occur in a ratio of 27:1 with a likelihood of 93.9%. In the same way, people bitten by ticks with anti-α-Gal IgG values greater than the cut-off value had a likelihood of suffering AGS of 35%.

Nowadays, two hypotheses have been proposed for explaining the sensitization to α-Gal and posterior allergy development. One proposes that α-Gal antigen is present on salivary proteins [44]. After biting, the α-Gal glycoproteins are presented to antigen-presenting cells (APCs) and B-lymphocytes in the context of Th2 cell-mediated immunity [44]. The second hypothesis implies the presence of immunomodulatory factors in tick saliva, such as PGE_2_ that triggers immunoglobulin class switching to anti-α-Gal IgE-producing B cells from preexisting mature B-cell clones producing anti-α-Gal IgM and/or IgG [45]. Oliveira et al. (2011) demonstrated the presence of non-protein molecules in tick saliva with potent immunomodulatory properties. Among these molecules, PGE2 was found in several tick species from major genera, such as *Ixodes* [64]. Gao et al. (2016) demonstrated that PGE2 promotes IgE production in vivo contributing to asthma development [63]. Specifically, PGE2 induces a class switch recombination on mature B cells [65]. Cabezas-Cruz et al. (2019) postulated that tick salivary PGE2 triggers Ab class switching in mature B cells, increasing the levels of anti-α-Gal IgE Abs [20]. Given that α-Gal exposure alone does not induce an IgE response [43], our results may be supported by the second hypothesis because all the AGS patients and risk-population individuals bitten by ticks demonstrated sIgG values to α-Gal statistically different from the atopic and healthy populations. Our data demonstrated that tick-bitten subjects present IgG Abs to α-Gal greater than subjects who were not bitten by ticks. From a statistical point of view, there seems to be a direct relationship between the levels of IgG and the possibility of developing AGS. 

However, our study has some limitations, such as the characteristics of the control selected populations and the relative low number of AGS patients included in the study. In following studies, it would be convenient to take into account the anti-α-Gal IgG subclass studied and the blood group of the individuals [41,46,47]. Finally, other characteristics of the individuals, such as age, the diet composition or the presence of intestinal parasites, could affect the composition of the microbiota and, therefore, the normal levels of anti-α-Gal Abs [65].

## 5. Conclusions

The relative levels of specific IgG against α-Gal can be quantified by an automated system using relative standardized calibrators defined for specific IgG. This quantification allowed us to establish a cut-off point for this parameter. According to the statistical results obtained, the quantification of IgG against α-Gal in subjects bitten by ticks could be used as a prognostic marker for developing mammalian meat allergy.

## Figures and Tables

**Table 1:** Clinical data on the AGS-patients.

Patient	Gender	Total IgE (kU_A_/L)	IgE Specific to α-Gal [kU_A_/L]	Clinical Symptoms
1	m	738	>100	Recurrent Urticaria
2	m	569	>100	Anaphylaxis
3	m	452	>100	Anaphylaxis
4	f	325	>100	Recurrent Urticaria
5	m	523	91.3	Recurrent Urticaria
6	m	238	79.9	Anaphylaxis
7	m	267	78.7	Anaphylaxis
8	m	461	76.0	Anaphylaxis
9	m	269	63.7	Anaphylaxis
10	m	230	54.4	Anaphylaxis
11	m	91.7	45.0	Acute Urticaria
12	m	671	41.6	Recurrent Urticaria
13	m	102	19.4	Recurrent Urticaria
14	m	139	16.8	Acute Urticaria
15	m	311	12.3	Anaphylaxis

**Table 2:** Characteristics of the risk-population group (*n* = 46) recorded by survey. Results are expressed as percentage (%) of total number of participants.

		Percentage (%)
	Male	34
	Female	66
Demographic data	Age (20–60 yrs.)	90
	Urban	83
	Rural	17
	Dog	41
Animal contact	Cat	24
	Others	20
Daily meat consumption		78
Meat-consumption related symptoms	None	90
	Intolerance	9
	Lyme	10
Chronic diseases	Asthma	10
	Fibromyalgia	4
	Arthritis	4
	Others	9

**Table 3:** Prevalence of sensitization in the risk and atopic population measured by ImmunoCAP ISAC. Results are expressed as percentage (%) of total number of participants.

		**Percentage (%)**	
**Allergen**	**Main Source of Allergen**	**Risk Population (*n* = 46)**	**Atopic Population (*n* = 64)**
Phl p 1	Grass pollen	15	53.1
Ole e 1	Olive tree pollen	8.6	37
Cup a 1	Cypress tree pollen	4.3	34
Cry j 1	Japanese cedar pollen	2.1	26
Bet v 2	Birch pollen	4.3	21.8
Der 1	Mites Group 1 allergens	30	30
Der 2	Mites Group 2 allergens	30	37.5
Der p 10	Mites Tropomyosin	0	4.6
Alt a 1	*Alternaria alternata*	0	12.5
Asp f 1	*Aspergillus fumigatus*	0	7.8
Pru p 3	Apricot	0	20
Cor a 8	Hazel	0	7.8
Act d 1	Kiwifruit	0	7.8
Pen m 1	Shrimp	0	4.6
Gad c 1	Egg	0	4.6
Fel d 1	Cat (uteroglobin)	13	26
Fel d 4	Cat (lipocalin)	6.5	6.25
Can f 1	Dog (lipocalin)	4.3	4.6
Mus m 1	Mouse (lipocalin)	2.1	6.2
Equ c 1	Horse (lipocalin)	0	3.1
Api m 1	Bee venom (phospholipase A2)	0	1.5
Pol d 5 (array)	Wasp venom (Antigen 5)	4.3	0
Mux F3 (array)	Carbohydrates (CCDs)	3.7	0

## Data Availability

The data presented in this study are available in the article.
